# Supplementary material for: Optogenetic control of pheromone gradients and mating behavior in budding yeast
Source: Life Sci Alliance. 2025 Apr 11;8(6):e202403078. doi: 10.26508/lsa.202403078 (PMC11992364; doi:10.26508/lsa.202403078)
Supplement: Supplementary file 2 [file LSA-2024-03078_TableS1.pdf]

Name	Background	Mating type	Relevant genotype	Alias / Description	Source
WT MATa	BY4741	*MAT*a	MATa; his3D1; leu2D0; met15D0; ura3D0	**Wild type *MAT*a**	Euroscarf
WT MATα	BY4742	*MATα*	MATα; his3D1; leu2D0; lys2D0; ura3D0	**Wild type *MATα***	Euroscarf
MH7	BY4742	*MATα*	[PC120]:MFα1 [PC120]:MFα2 his::EL222	Native pheromone gene dosage opto-α strain	This study
MH10	BY4741	*MAT*a	[PC120]:Bar1 his::EL222	**Opto-Bar1 strain**	This study
MH11	BY4742	*MATα*	[PC120]:MFα1 [PC120]:MFα2 his::EL222 HO::PC120-MFα1	**Opto-α strain**	This study
MH16	BY4742	*MATα*	[PC120]:MFα1 [PC120]:MFα2 his::EL222 HO::PC120-MFα1 HTB2::mApple-kanMX	**Opto-α*** strain. An opto-α strain carrying a mApple-HTB2 fusion as an unclear fluorescent marker	This study
CE4	BY4741	*MAT*a	[PC120]:MFA1 [PC120]:MFA2 his::EL222	Native pheromone gene dosage opto-a strain	This study
MH21	BY4741	*MAT*a	[PC120]:Bar1 his::EL222 HTB2::mApple-kanMX	An opto-Bar1strain carrying a mApple -HTB2 fusion as a nuclear fluorescent marker	This study
yAA198	SEY6210a	*MAT*a	ura3::pAA35[PFUS1-Ubi(I)-sfGFP-3'FUS1URA3] aga2Δ::klTRP1	Non-agglutinating α-factor biosensor	Alexander Anders, MPI, Marburg
yAA156-	SEY6210	*MATα*	ura3::pAA35[PFUS1-Ubi(I)-sfGFP-3'FUS1	a-factor biosensor	Alexander Anders, MPI,

1			URA3]		Marburg
yAA24-1	SEY6210a	*MAT*a	ura3::pAA35[PFUS1-Ubi(I)-sfGFP-3'FUS1 URA3]	α-factor biosensor	Alexander Anders, MPI, Marburg
yAA28	SEY6210a	*MAT*a	bar1Δ::kanMx6 ura3::pAA35[PFUS1-Ubi(I)-sfGFP-3'FUS1 URA3]	Bar1Δ α-factor biosensor	Alexander Anders, MPI, Marburg
MH26	BY4742	*MAT*α	WT *MAT*α + mVenus Nuclear Tag	WT *MAT*α carrying a mApple-HTB2 fusion as a nuclear fluorescent marker	This study
yPH471	BY4741	*MAT*a	HO : pC120-SUC2-P2A-Venus - HIS3::(pPGK1-VP16-EL222-tCYC1) HTB2::mApple-kanMX	Biomask control for half-domain assay	Ref[2]

Supplementary Table 1. *Saccharomyces cerevisiae* strains used in this study.
