# Supplementary material for: Optogenetic control of pheromone gradients and mating behavior in budding yeast
Source: Life Sci Alliance. 2025 Apr 11;8(6):e202403078. doi: 10.26508/lsa.202403078 (PMC11992364; doi:10.26508/lsa.202403078)
Supplement: Supplementary file 3 [file LSA-2024-03078_TableS2.pdf]

Supplementary table 2. Oligonucleotides and guide RNA sequences used in this study.

Name	Sequence	Description
oPH_920	CTAGCTCTAAAACTTCATTGACATCACTAGAGA	short guide for CRISPR insertion of PC120 upstream of MFA2
oPH_921	CTAGCTCTAAAACCTCATTAATTCATTTCTGGC	short guide for CRISPR insertion of PC120 upstream of MFA1
oPH_922	TATAGTTGTCTTTCTTTTCAGAGGATTTATCCTTCT GAGTGGCTTGTGTGGAAGCAGTGGTGATCGGTTG CATAGATTTTAGCGGCCGCG	Reverse primer for the amplification of PC120 from plasmid pYTK097 with overhangs for MFA2 promoter replacement
oPH_923	GCTGTTGCATTACCACGTAATTTTGTATATAAATA TCTGATAAATAACCATTTTATTTCCATCCACTTCT TTATCGCCGGGTACGTGAGT	Forward primer for the amplification of PC120 from plasmid pYTK097 with overhangs for MFA2 promoter replacement (long version)
oPH_924	GCATGTATTTACCTATTCGGGAAATTTACATGAC ATGGATGCCATAAGGAACGAAAATGAAACATGC ATGTTATCGCCGGGTACGTGAGT	Forward primer for the amplification of PC120 from plasmid pYTK097 with overhangs for MFA2 promoter replacement (short version)
oPH_925	TGATAATATAGTTGTCCTTCTTTTCACTGCTGGTCT TTTCTTTTGGAGCGGCGGTAGCGGTAGATGGTTG CATAGATTTTAGCGGCCGCG	Reverse primer for the amplification of PC120 from plasmid pYTK097 with overhangs for MFA1 promoter replacement
oPH_926	CCTACTGCTACGGTTGGCCCATACCTTTATTCTTT GTTCTTGTTACAAACGAGTGTGTAATTACCCAAA ATTATCGCCGGGTACGTGAGT	Forward primer for the amplification of PC120 from plasmid pYTK097 with overhangs for MFA1 promoter replacement

oPH_927	GATCTCTCTAGTGATGTCAATGAAGTTTTAGAGCTAG	Long guide for CRISPR insertion of PC120 upstream of MFA2
oPH_928	GATCGCCAGAAATGAATTAATGAGGTTTTAGAGCTAG	Long guide for CRISPR insertion of PC120 upstream of MFA1
oPH_775	ATT GAG CTT CTT TTC TTG AGG AGA GAT CCA ATT TGA AGT CGG AAT AAG ATT TGC TTT CAT TAG CGT AGG CTT ATC GCC GGG TAC GTG AGT	Forward primer for the amplification of PC120 from plasmid pYTK097 with overhangs for MF(Alpha)1 promoter replacement
oPH_776	TAG TGT TGA CTG GAG CAG CTA ATG CGG AGG ATG CTG CGA ATA AAA CTG CAG TAA AAA TTG AAG GAA ATC TCA TAG ATT TTA GCG GCC GCG	Reverse primer for the amplification of PC120 from plasmid pYTK097 with overhangs for MF(Alpha)1 promoter replacement
oPH_777	GAT CTA GCT TCT ACT GAA AAA CAG GTT TTA GAG CTA G	Long guide for CRISPR insertion of PC120 upstream of MF(Alpha)1
oPH_778	CTA GCT CTA AAA CCT GTT TTT CAG TAG AAG CTA	short guide for CRISPR insertion of PC120 upstream of MF(Alpha)1
oPH_783	GATCTCTTTACAGCGCAGAGACGAGTTTTAGAGCTAG	Long guide for CRISPR insertion of PC120 upstream of MF(Alpha)2
oPH_784	CTAGCTCTAAAACTCGTCTCTGCGCTGTAAAGA	short guide for CRISPR insertion of PC120 upstream of MF(Alpha)2
oPH_787	TCGGGAAACTCTATAGTTTTCTGCGTTTCAGTACGCAGTTGGGCGTGCTAAAGTTGTTTCCTAATTTGCTTATCGCCGGGTACGTGAGT	Forward primer for the amplification of PC120 from plasmid pYTK097 with overhangs for MF(Alpha)2 promoter replacement

oPH_788	TATCTTCATCGGAACTAGCAGTGACAGAAACGGCCGCTAAAATAAAAGTGAGAAAGGTAGAAATGAATTTCATAGATTTTAGCGGCCGCG	Reverse primer for the amplification of PC120 from plasmid pYTK097 with overhangs for MF(Alpha)2 promoter replacement
oPH_793	GATCTCCGACATCATGCTGAAACAGTTTTAGAGCTAG	Long guide for CRISPR insertion of PC120 upstream of BAR1
oPH_794	CTAGCTCTAAAACTGTTTCAGCATGATGTCGGA	short guide for CRISPR insertion of PC120 upstream of BAR1
oPH_795	AAAGCGCCGGTTCCTCTGACTCTAGAAGAACAAATTGACAATGTGTCGTTGAGATACGGCAACGAGTTGGTTATCGCCGGGTACGTGAGT	Forward primer for the amplification of PC120 from plasmid pYTK097 with overhangs for Bar1 promoter replacement
oPH_796	AAGCAGTAATGGTGTTAATAATCGCGAAACTCGCCAAAATAAGTTTCAAACAAAGATGATTAATTGCAGACATAGATTTTAGCGGCCGCG	Reverse primer for the amplification of PC120 from plasmid pYTK097 with overhangs for Bar1 promoter replacement
oPH_805	CCA CGA AAA GTT CAC CAT AAC TTC GAA TAA AGT CGC GGA AAA AAG TAA ACA GCT ATT GCT ACT CAA ATG ATT ATC GCC GGG TAC GTG AGT	Forward primer for the amplification of the PC120-MF(ALPHA)1 cassette from genomic DNA from strain MH7 with overhangs for insertion in the HO locus
oPH_811	TGG TTT TTT TCA TCC AAA ATA TTA AAT TTT ACT TTT ATT ACA TAC AAC TTT TTA AAC TAA TAT ACA CAT TGG CAT CAT AAT CAG GGA GTG	Forward primer for the amplification of the PC120-MF(ALPHA)1 cassette from genomic DNA from strain MH7 with overhangs for insertion in the HO locus
