# Supplementary material for: Optogenetic control of pheromone gradients and mating behavior in budding yeast
Source: Life Sci Alliance. 2025 Apr 11;8(6):e202403078. doi: 10.26508/lsa.202403078 (PMC11992364; doi:10.26508/lsa.202403078)
Supplement: Supplementary file 4 [file LSA-2024-03078_TableS3.pdf]

Supplementary Table 3. Fit parameters for gene expression and cell elongation response functions.

				**Best model fit***			
n	$cost$	C (EC$_{50}$)	λ_α (μm)	A_{gfp}	A_{elo}	b_{gfp}	b_{elo}
1	9660	0.485	798	$1.19 \cdot 10^{-1}$	26.7	$2.84 \cdot 10^{-3}$	2.74
2	5222	1.00	766	$7.54 \cdot 10^{-2}$	17.0	$1.67 \cdot 10^{-3}$	4.23
**3**	**5159**	**1.45**	**735**	**$6.28 \cdot 10^{-2}$**	**10.7**	**$8.63 \cdot 10^{-4}$**	**4.55**
4	5856	1.63	728	$5.94 \cdot 10^{-2}$	8.69	$5.10 \cdot 10^{-4}$	4.66
5	6543	1.64	715	$5.93 \cdot 10^{-2}$	8.11	$6.11 \cdot 10^{-4}$	4.72
6	7011	1.58	683	$5.98 \cdot 10^{-2}$	7.92	$1.01 \cdot 10^{-3}$	4.76
7	7291	1.56	659	$5.99 \cdot 10^{-2}$	7.73	$1.29 \cdot 10^{-3}$	4.77

* Each row presents the best fit with n equal to the value in the first column, fits are obtained by optimizing the *cost* value calculated by the *scipy.optimize.least_squares* python library function at fixed n. The best fit for both models was chosen to minimize the *cost* across the whole column, and is presented in bold letters. Unspecified dimensions are arbitrary units.
